# Supplementary figures and images for: Polyvinylalcohol-carbazate (PVAC) reduces red blood cell hemolysis
Source: PLoS One. 2019 Dec 6;14(12):e0225777. doi: 10.1371/journal.pone.0225777 (PMC6897416; doi:10.1371/journal.pone.0225777)

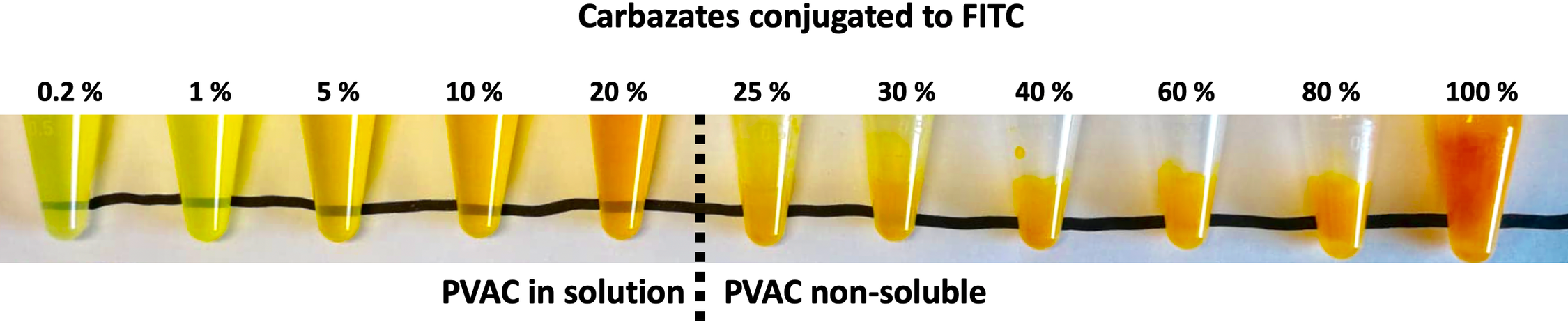

Supplement: S1 Fig — (TIF) [file pone.0225777.s001.tif]

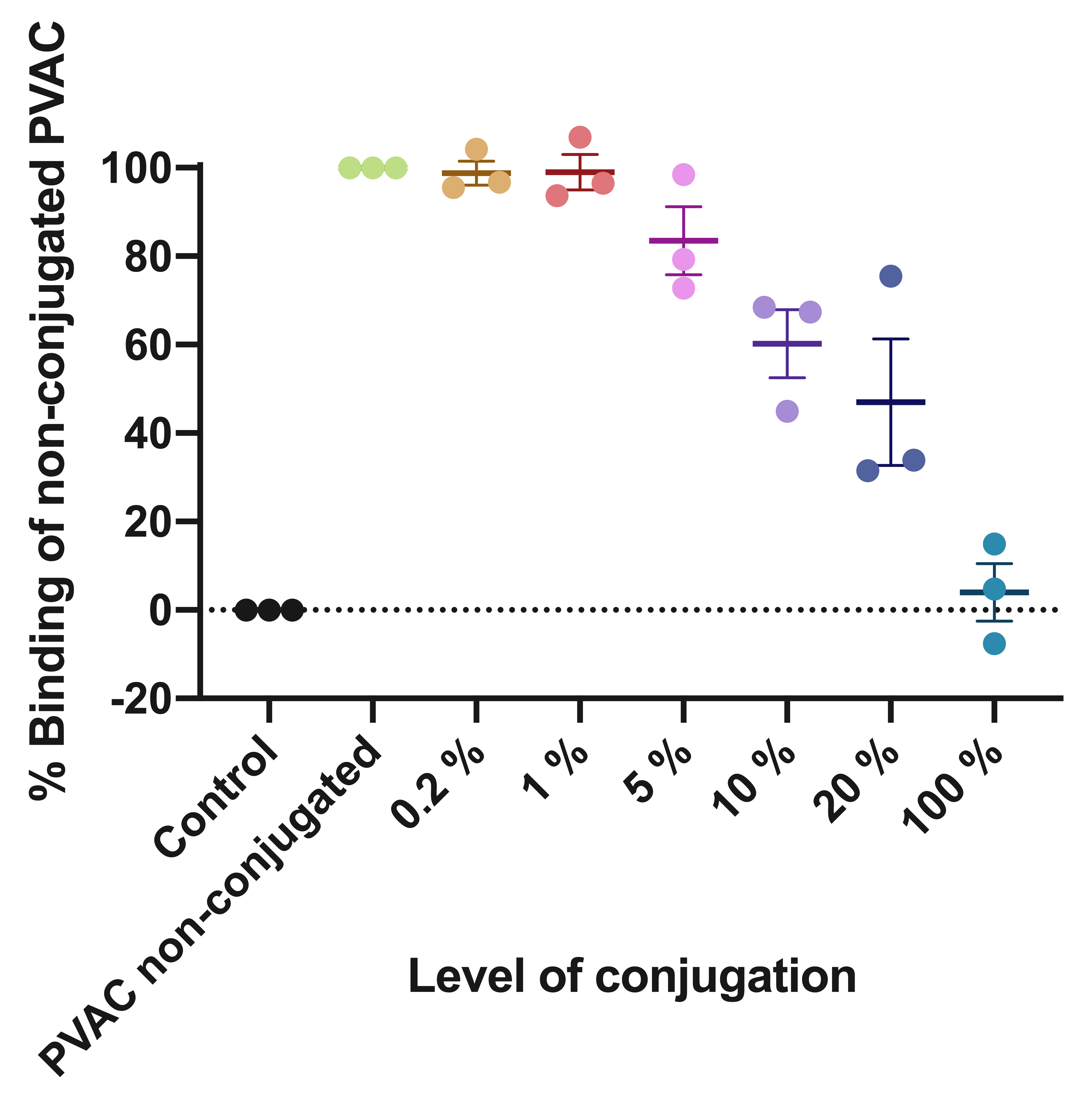

Supplement: S2 Fig — (TIF) [file pone.0225777.s002.tif]

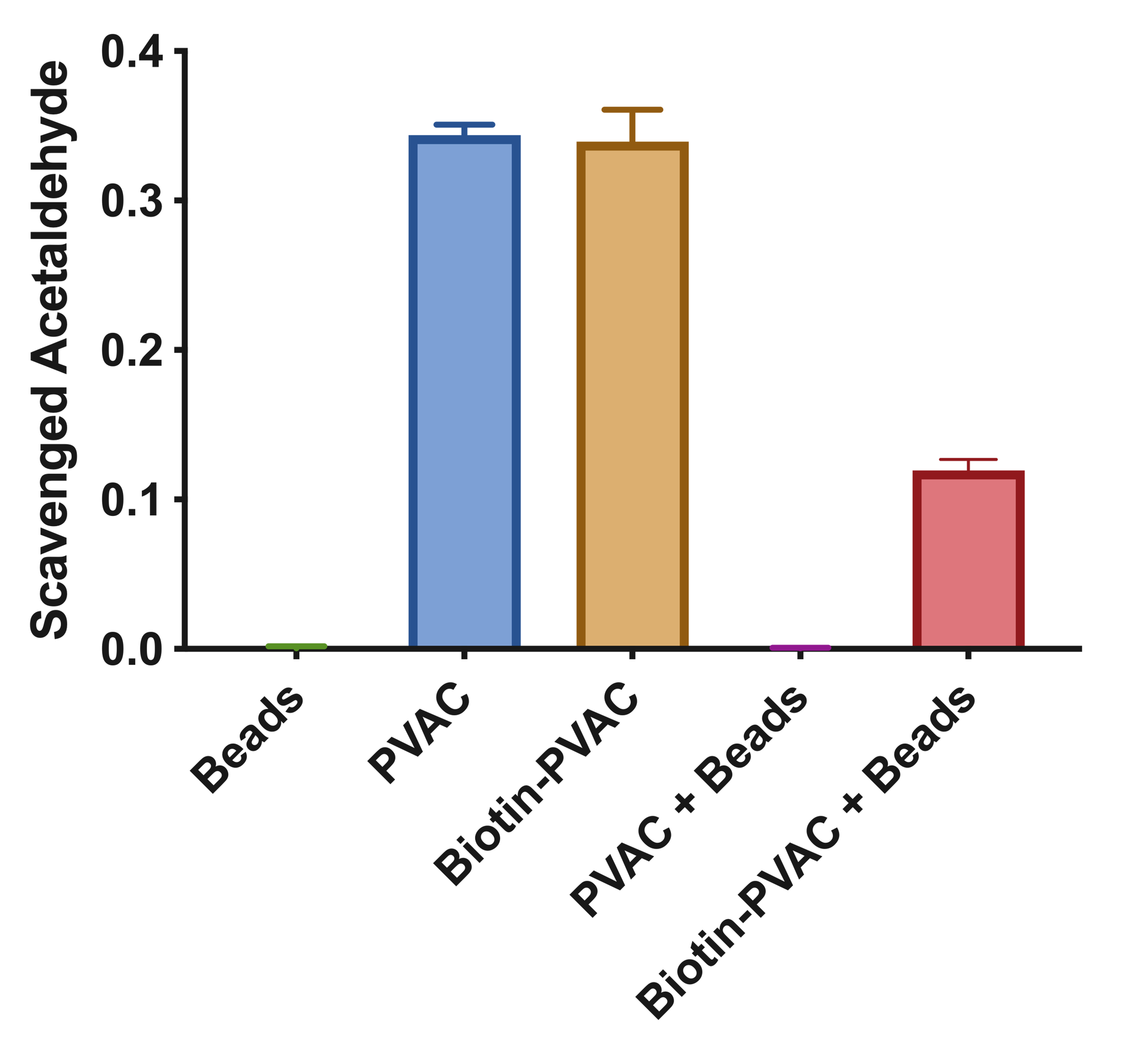

Supplement: S3 Fig — (TIF) [file pone.0225777.s003.tif]

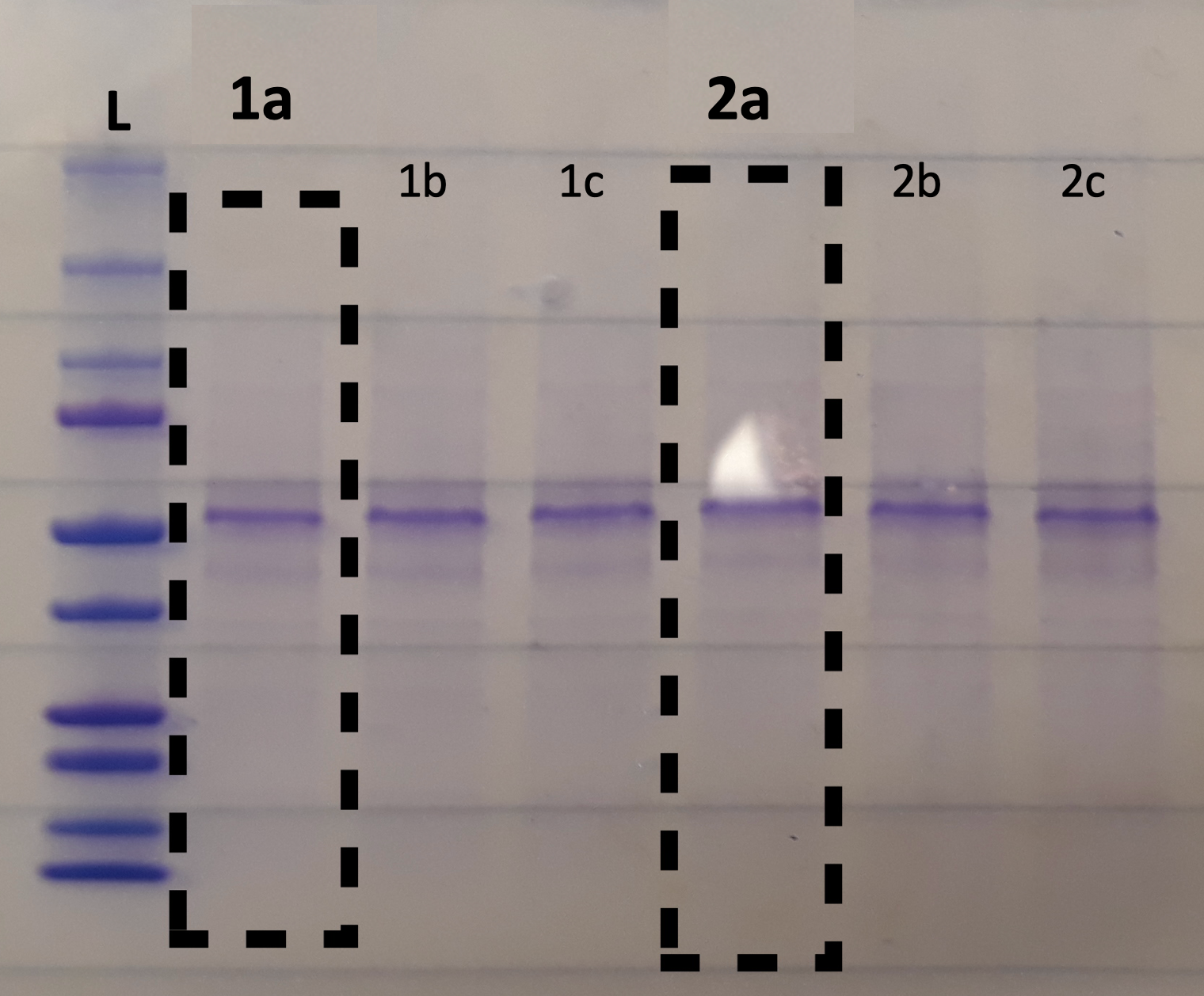

Supplement: S4 Fig — (TIF) [file pone.0225777.s004.tif]

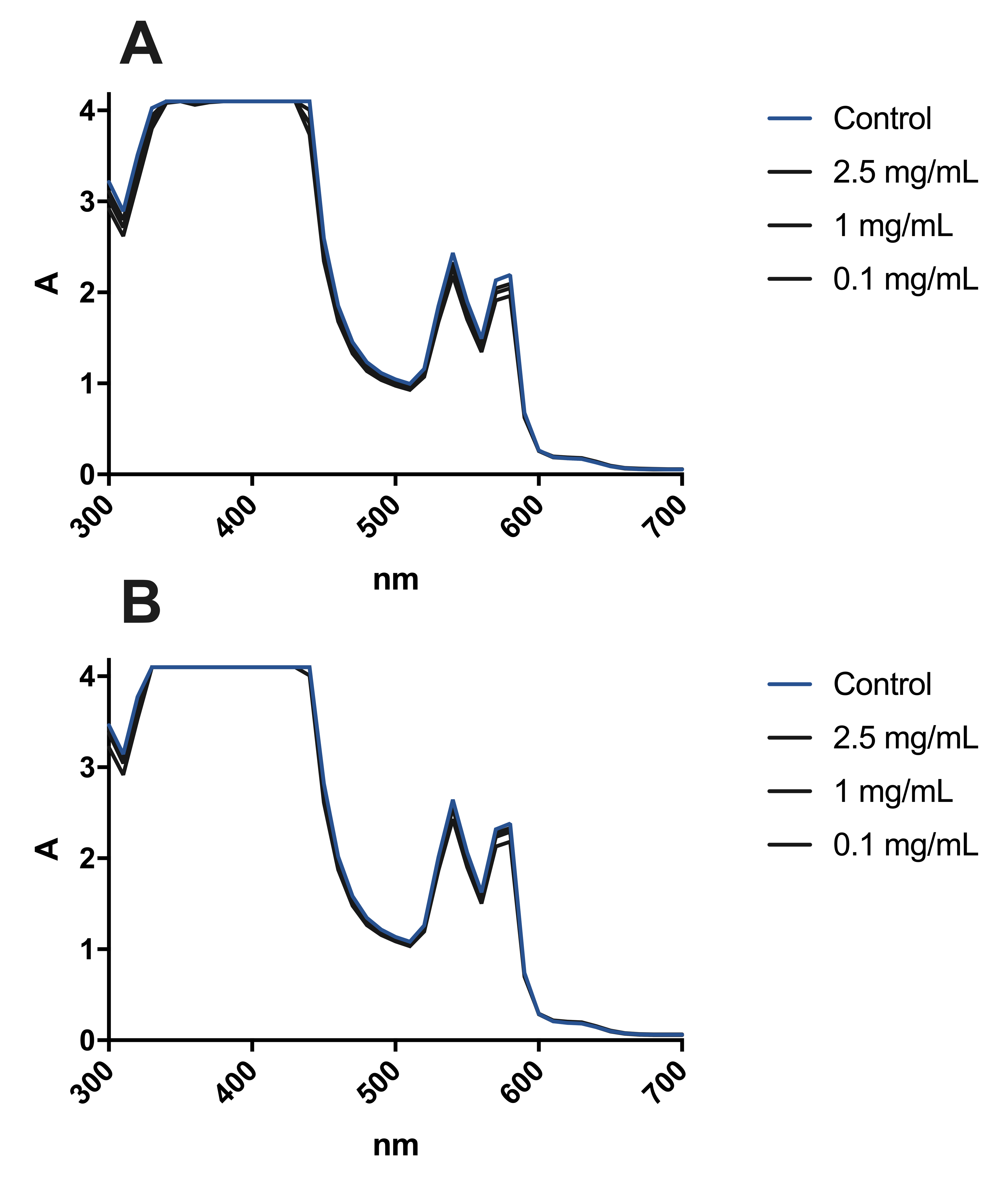

Supplement: S5 Fig — (TIF) [file pone.0225777.s005.tif]

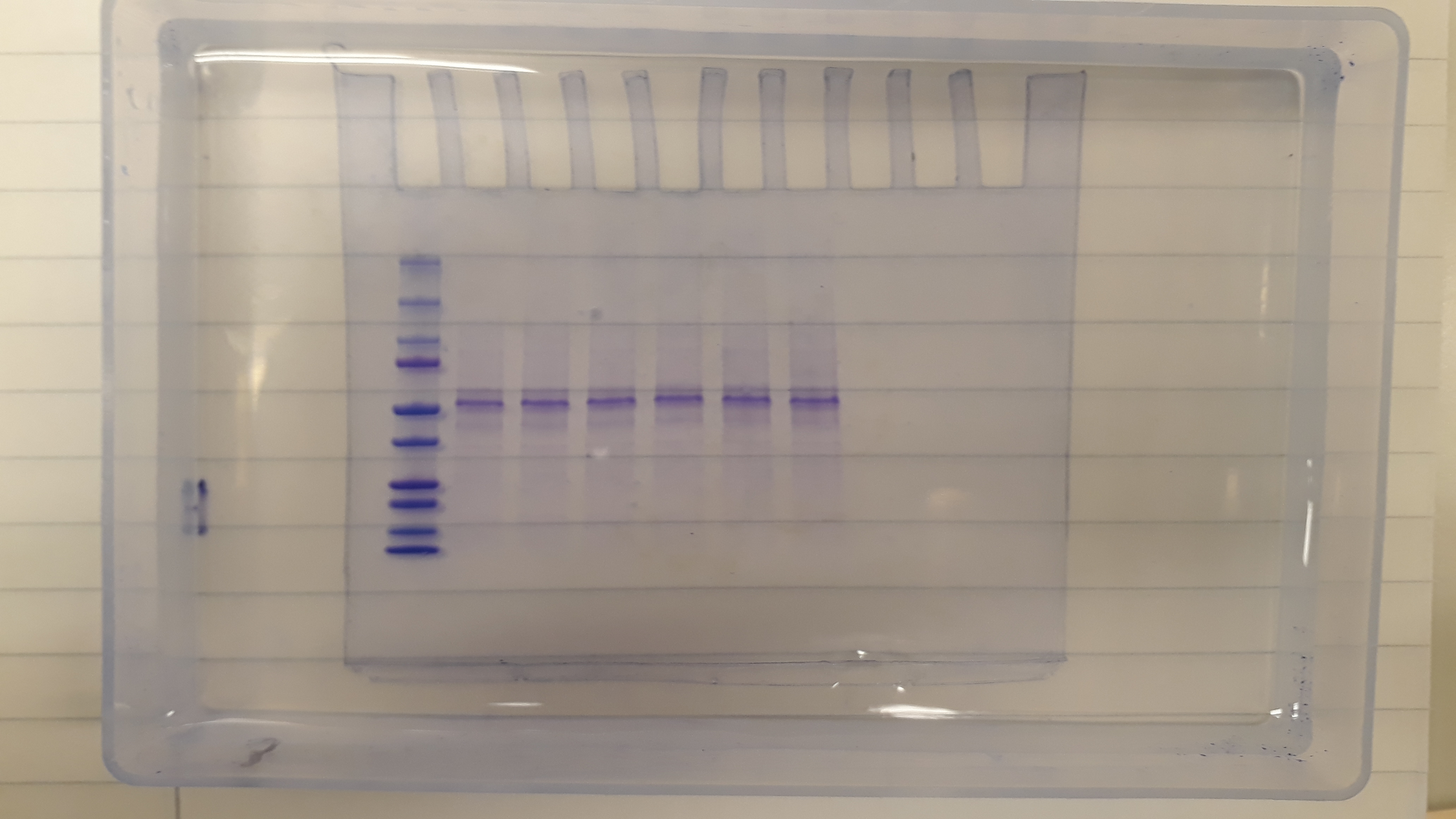

Supplement: S6 Fig — (TIF) [file pone.0225777.s006.tif]
