# Supplementary material for: Polyvinylalcohol-carbazate (PVAC) reduces red blood cell hemolysis
Source: PLoS One. 2019 Dec 6;14(12):e0225777. doi: 10.1371/journal.pone.0225777 (PMC6897416; doi:10.1371/journal.pone.0225777)
Supplement: S1 File — (DOCX) [file pone.0225777.s007.docx]

Supplementary material

# **Materials and methods**

## **Chemical modifications**

To find an appropriate level of conjugation balancing functionality of the polymer and detection different levels were tested. The carbazate content in PVAC is roughly 1200 μmol/mg and to test an appropriate level of conjugation, PVAC (1 mg, in 1 mL MQ) was conjugated to FITC (according to table 1, in DMSO 0.1 mL) by mixing (60 min).

**Table 1.** **The different levels of conjugation used for PVAC and FITC.**

| FITC (µmol) | 2.4 |  | 12 | 60 | 120 |  | 240 | 300 | 360 | 480 | 720 | 960 | 1200 |  |
| --- | --- | --- | --- | --- | --- | --- | --- | --- | --- | --- | --- | --- | --- | --- |
| Conjugation (%) | 0.2 |  | 1 | 5 | 10 |  | 20 | 25 | 30 | 40 | 60 | 80 | 100 |  |
| PVAC (carbazate, µmol) | 0.001 |  | 0.005 | 0.025 | 0.05 |  | 0.1 | 0.13 | 0.15 | 0.2 | 0.3 | 0.4 | 0.5 |  |

The different conjugations were inspected visually to check if PVAC remained in solution or if there was precipitation formed. Afterwards the remaining clear solution was checked for its ability to neutralize acetaldehyde using the Megazyme assay, as outlined in the main document. FITC-PVAC conjugate formed was also exited at 485 nm and emission was registered at 528/20 nm using a Synergy HTX plate reader (BioTek).

## **Immunoprecipitation (IP) and mass spectrometry (MS)**

#### Chemicals and reagents

Acetonitrile (ACN), acetic acid (HAc), formic acid (FA), ammonium bicarbonate (NH_4_HCO_3_), trifluoroacetic acid (TFA), iodoacetamide (IAA) and dithiothreitol (DTT) were purchased from Sigma Aldrich (St. Louis, MO, USA). Trypsin (MS grade; Promega, Mannheim, Germany) was used. Ultrapure water was prepared by Milli-Q water purification system (Millipore, Bedford, MA, USA).

RBCs were incubated with biotin-PVAC at a concentration of 100 μg/mL for 30 min with shaking. The RBCs were then washed twice in ice-cold PBS before lysis with Pierce™ IP Lysis Buffer (Thermofisher) with added protease inhibitors for 5 min on ice. Dynabeads™ MyOne™ Streptavidin C1 concentrate (100 μL) was washed twice in ice-cold PBS (1 mL) using a handheld magnet. After washing lysate (1 mL) was added to the beads and the mixture was incubated for 1 h with gentle rotation at 2 ℃. The beads were separated from the mixture using a magnet and then washed four times in PBS (1 mL) using the magnet. After washing the pH was lowered to < 2 with HCl (1 M) to hydrolyse the bond between PVAC and the proteins releasing them from the beads. The beads were removed with the magnet and the protein content was determined in the remaining fluid using Pierce™ Coomassie Plus (Bradford) Assay Kit (Thermofisher) according to manufacturer’s instructions. The protein solution was then frozen at -80 ℃. The same protocol as outlined above was repeated with the addition of none-conjugated PVAC and just PBS as controls. The acetaldehyde binding was assayed as outlined in the manuscript.

The protein solutions were thawed and 5-10 μg was set in to the wells of a SDS-PAGE 4-20% gel, Precision Plus Protein™ Standards Kaleidoscope (BioRad, Hercules, CA, USA) was used as a ladder. The gel ran at 100 V for 45 min. The gel was stained with Imperial™ Protein Stain (Thermofisher) according the manufactures instructions. The lanes were cut and placed in 1.5 mL test tubes filled with DI water and then transferred to the MS facility.

#### In-gel tryptic digestion of proteins.

For in-gel digestion, selected gel bands were cut into small pieces, neutralized with 25 mM NH_4_HCO_3_. A volume of 150 μL of ACN was added to the gel pieces and the samples were incubated by agitation for 5 min at room temperature and the liquid was then discarded. This step was repeated two times until most Coomassie stain was removed. The gel pieces were then dried in Speedvac system ISS110 (Thermo Scientific, Waltham, MA, USA) for 10-15 min and exposed to DTT reduction and IAA alkylation. Thereafter the proteins were digested by sequencing grade modified trypsin at a concentration of 12.5 ng/µL in 25 mM NH_4_HCO_3_ overnight at 37^o^C. The peptides were extracted by sonication in 60% ACN and 5% FA. Finally, the extracted peptides were completely dried and redissolved in 20 μL 0.5% TFA and desalted on Pierce™ C18 spin columns (Thermo Scientific, Rockford, IL 61105 USA) using the following schedule: the column was first wetted in 2×200 μL of 50% ACN and equilibrated with 2×200 μL 0.5% TFA. The tryptic peptides were adsorbed to the media using five repeated cycles of loading. The column was washed using 4×200 μL 0.5% TFA and finally the peptides were eluted in 2×30 μL 70% ACN. After desalting, the eluate was vacuum centrifuged to dryness and re-dissolved in 30 μL 0.1% FA and further diluted 4 times more prior to nano-LC-MS/MS.

NanoLC-MS/MS analysis
The nanoLC-MS/MS experiments were performed using a Q Exactive Orbitrap mass spectrometer (ThermoFisher Scientific, Bremen, Germany) equipped with a nano electrospray ion source. The peptides were separated by C18 reversed phase liquid chromatography using an EASY-nLC 1000 system (Thermo Fisher Scientific). A set-up of pre-column and analytical column was used. The pre-column was a 2 cm EASYcolumn (ID 100 µm, 5 µm particles) (Thermo Fisher Scientific) while the analytical column was a 10 cm EASY-column (ID 75 µm, 3 µm particles, Thermo Fisher Scientific). Peptides were eluted with a 90 min linear gradient from 4% to 100% acetonitrile at 250 nL min-1. The mass spectrometer was operated in positive ion mode acquiring a survey mass spectrum with resolving power 70,000 (full width half maximum), m/z 400-1750 using an automatic gain control (AGC) target of 3×10^6^. The 10 most intense ions were selected for higher-energy collisional dissociation (HCD) fragmentation (25% normalized collision energy) and MS/MS spectra were generated with an AGC target of 5×10^5^ at a resolution of 17,500. The mass spectrometer worked in data-dependent mode.

#### Data analysis

The acquired data (.RAW- files) were processed by Proteome Discoverer software (Thermo Scientific, version [nr 1.4.1.14]) using the Sequest algorithm against the UniProt human database (release September 2017). The following parameters were used for data processing: maximum 10 ppm and 0.02 Da error tolerances for the survey scan and MS/MS analysis, respectively, trypsin as digesting enzyme, carbamidomethylation of cysteins as fixed modification, oxidation of methionine as variable modification, maximum of two miss cleavages sites. The search criteria for protein identification were set to at least two matching peptides per protein.

# **Results**

## **Chemical modification**

When the level of conjugation was increased to more than 20% of carbazate groups PVAC started to precipitate. (Fig 1) Conjugation must therefore be lower than 20% to preserve the solubility in water of PVAC.

S Fig 1.

**Fig 1.** Picture captured with a mobile cell phone (Samsung A3). Eppendorf tubes containing mixture of FITC and PVAC placed against a white background. A black line was drawn on the paper using a marker, when precipitation is formed the line is no longer visible through the clear solution. Dotted line represents between which two concentrations precipitation started to form.

As the level of conjugation increases the scavenging potential of PVAC is reduced. At 5% conjugation the scavenging potential starts to be affected with roughly 80% left. At 10% conjugation roughly 50% of the scavenging potential is left and after 100% of carbazate groups are conjugated no acetaldehyde can be bound. (Fig 2) To preserve the binding capabilities of PVAC the two levels of conjugation not resulting in a reduced aldehyde scavenging potential (0.2 and 1%) were further tested for their emission peaks. Both resulted in a strong signal so the lowest level tested was chosen for the experiments in the manuscript.

S Fig 2.

**Fig 2.** Graph depicting the acetaldehyde neutralizing potential of PVAC after conjugation to FITC at different levels. Y-axis is the neutralization potential as % of non-conjugated PVAC control, x-axis is the different levels of conjugation, experiment was carried out in three series.

## **MS and IP**

PVAC modified with biotin or unmodified was assayed for its acetaldehyde binding capabilities with no difference in signal meaning the modified version remains functional. Beads incubated with biotin-PVAC, PVAC or just PBS were washed and then analyzed for their acetaldehyde binding capacity. The results indicate that PVAC has no affinity for the beads without biotin modification and that the molecule remains functional with regards to a known ligand when immobilized on the beads. (Fig 3)

S Fig 3.

**Fig 3.** Y-axis indicates scavenged acetaldehyde in the assay as a reduction in absorbance at 340 nm compared to control. Biotin-PVAC did not differ from non-modified PVAC in its ability to scavenge a known ligand meaning that the function is mostly intact after modification. Beads were incubated with biotin-PVAC, PVAC or, just PBS and then washed using a magnet. A reduction in signal was only seen with the biotin-conjugated PVAC acting as evidence that the beads to interact with biotin-PVAC and that PVAC remains functional on the surface of the beads.

No difference was seen in the pattern of isolated proteins under control conditions or in the presence of PVAC. (Fig 4) Two bands were still cut to identify the proteins and check for possible differences in the pattern of isolated proteins.

S Fig 4.

**Fig 4.** Gel picture captured with a mobile phone (Samsung A3). Ladder (Precision Plus Protein™) in the left most lane. Lanes marked with 1 marks the samples treated with biotin-PVAC while lanes marked with 2 the controls treated with PBS. Samples from three different donors (a, b and, c) were used. Dotted lanes were cut and sent for analysis with MS.

**Table 1.** Top 5 scored proteins identified via MS.

| Biotin-PVAC | | | Vehicle (PBS) | | |
| --- | --- | --- | --- | --- | --- |
| Accession | **Protein ID** | **Score** | **Accession** | **Protein ID** | **Score** |
| P11277 | Spectrin beta chain, erythrocytic | 2438,31 | P11277 | Spectrin beta chain, erythrocytic | 1786,64 |
| P02549 | Spectrin alpha chain, erythrocytic | 2175,66 | P02549 | Spectrin alpha chain, erythrocytic | 1684,81 |
| H0YBS0 | Ankyrin-1 | 914,93 | H0YBS0 | Ankyrin-1 | 1086,39 |
| P04264 | Keratin, Type II | 515,45 | P04264 | Keratin, Type II | 454,92 |
| P11171 | Protein 4.1 | 419.97 | P11171 | Protein 4.1 | 412,02 |

A total of 136 unique proteins were identified in the sample treated with PVAC, however, most of these were also found in the control setting without PVAC added. The top five scoring proteins can be found in table 1 and are representative of the very similar profiles of the isolated proteins. For the full list of isolated proteins please see the raw data.

## **Accurate hemoglobin measurements**

As described in the manuscript no difference in the absorbance spectra for hemoglobin was noted with the addition of PVAC, thus PVAC does not seem to interfere with the way hemoglobin was measured in the study.

S Fig 5.

**Fig 5 a,b.** The absorbance spectra of hemoglobin in 10 nm increments. The spectra was registered with or without the addition of PVAC in three different doses. **(a)** The spectra after a single dose of PVAC. **(b)** The spectra after an additional dose of PVAC.
