# Supplementary material for: Polyvinylalcohol-carbazate (PVAC) reduces red blood cell hemolysis
Source: PLoS One. 2019 Dec 6;14(12):e0225777. doi: 10.1371/journal.pone.0225777 (PMC6897416; doi:10.1371/journal.pone.0225777)
Supplement: Attachment — Submitted filename: Response to reviewers.docx [file pone.0225777.s011.docx]

**Response to reviewers**

**1. Content Feedback**

1.1. Page 3, Line 49: The terminology “such anti-hemolytic agents” should not be used here, unless these agents, or the notion thereof, are directly introduced in the preceding sentence.

**We agree with the comment and have rephrased the statement accordingly.**

1.2. Page 3, Line 58: It is stated that hemolysis shortens the possible length of storage. This statement may be bolstered with specification of contemporary RBC storage parameters and how these are contingent upon in-bag hemolysis.

**We agree that this would strengthen the introduction to our work. We have expanded the introduction to include more information about the storage lesion including the role of carbonylation and how it relates to contemporary RBC storage.**

1.3. Page 3, Line 59: How does carbonylation occur? Are there mechanisms in vivo that prevent carbonylation and/or its propagation?

**Please see answer to 1.2**

1.4. Page 5, Line 86: Please expand upon “desired buffer and concentration.” While covered later in the methods, expanding upon this (i.e. listing the buffer options) would make this section more understandable and enhance cohesiveness of the methods section as a whole. Components should at least be noted for frame of reference.

**We appreciate the comment by the reviewer and we have revised accordingly.**

1.5. Page 5, Line 87: The sentence states “the whole blood chamber model,” but this model has not yet been introduced. In the same vein of 1.4, adding detail here will help with understandability of the section as a whole.

**We agree that this sentence was out of context and it has been rephrased to reference biocompatibility.**

1.6. Page 5, Line 88: By which metrics was donor health assessed?

**“Healthy donors” are assessed using a screening process after which their blood donations can be used for clinical transfusion. This is a rigorous process that includes medical and laboratory assessments that determine the health status of the donor and that follow internationally established standards. A brief statement has been added in the section on RBC preparation.**

1.7. Page 5, Line 95: Why was a second addition of PVAC employed for only the warm storage group, and not the cold storage group, following one week?

**The rationale for multiple additions in the warm storage group is that we expected a greater degree of hemolysis during warm storage, which was based on knowledge from laboratory and clinical practices utilizing cold storage to limit hemolysis. The lowering of temperature leads to a slower? metabolism which lowers oxidative stress and lysis of RBC. This was further confirmed in our study where the increase in temperature increased the hemolysis. The materials and methods has been edited to explain the rationale.**

1.8. Page 6, Line 109: Please specify which signature corresponds to 540 nm (Q-band) and, thus, why absorbance at this wavelength was us used in analyses.

**Depending on the oxidative state of hemoglobin the absorbance spectrum changes. RBC stored in the presence of oxygen are generally in the state of oxyhemoglobin which has an absorbance peak of approximately 540 nm. We confirmed this in our study by measuring the absorbance spectrum of hemoglobin between 300 and 700 nm (in sup Fig 5). We subsequently determined that 540 nm was an appropriate wavelength for detection also in our investigations. In materials and methods we have added a paragraph to clarify the wavelengths at which hemoglobin was measured.**

1.9. Page 8, Line 158: Please introduce what C3a is and why it is significant for assessment.

**In materials and methods we have added a description of C3a, which is a molecule formed in the complement cascade that signals inflammation. In the whole blood model, increasing levels of C3a is an appropriate marker for general complement activation.**

1.10. Page 11, Line 226: What is DNR 2008/264?

**This is an identification number used by the Swedish Regional Ethical Review Board. We have clarified this in our ethical statement in the materials and methods and added the statement to our supporting information.**

1.11. Page 13, Line 284: For figure four, the Y-axis reads “free aldehydes (%),” while the caption reads “free acetaldehyde.” Given that other aldehydes (i.e. MDA) may be present in the sample with storage age, perhaps the Y-axis should specify “acetaldehydes.”

**We appreciate this astute observation and have clarified that we are indeed measuring not only acetaldehyde but in fact all free aldehydes. The method used relies on the enzyme aldehyde dehydrogenase and measures reduction of the substrates for the enzyme; we have also tested and confirmed that the method works with for example MDA.**

1.12. Page 14, Line 332: What additional stresses are present at room temperature to cause additional hemolysis? Please specify. This point may further be driven by introducing contemporary storage parameters and why cold storage is instituted (i.e. how hemolysis mechanisms are altered at lower temperatures) in the background section.

**We have added additional information in the introduction/background regarding contemporary storage parameters. Please also see our response to point 1.2.**

**2. Grammatical Edits**

2.1. Page 3, Line 44: Prior to using the acronym “RBCs” in the body text, please introduce this in parentheses following “red blood cells.” In this section, “RBCs” is used, but throughout the body “RBC” is later used to refer to plural erythrocytes. These uses need be altered to “RBCs” for clarity.

**We have changed the abbreviation so that only RBCs is used throughout the manuscript, since we only refer to red blood cells in the plural form.**

2.2. Page 3, Line 46: Please change “haemolytic” to “hemolytic” for consistency with other uses of hemolysis throughout the manuscript.
2.3. Page 3, Line 47: A comma needs to be added prior to “where toxins lyse the RBC” for readability.
2.4. Page 3, Line 49: Please change “haemolytic” to “hemolytic” (see 2.2).
2.5. Page 4, Line 82: Both uses in of “is” here should be changed to “are.”
2.6. Page 6, Line 108: Minor typo of “minuntes.”
2.7. Page 8, Line 172: Should “immune precipitation” read “immunoprecipitation” here?
2.8. Page 12, Line 257: Please change the comma following “spectra” to a period.
2.9. Page 13, Line 280: Please change the comma following “acetaldehyde” to a period.
2.10. Page 13, Line 290: Please begin the sentence with “blood compatibility,” or some other terminology to complete this sentence.
2.11. Suppl. Line 6: “a appropriate” should be changed to “an appropriate.”
2.12. Suppl. Line 28: Please change “ice cold” to “ice-cold” for consistency with previous usage.
2.13. Suppl. Line 119: “none-modified” should be change to “unmodified.”
2.14. Suppl. Line 149: For consistency with other uses of “hem-“ in the main text, please change “haemoglobin” to “hemoglobin.”
2.15. Suppl. Line 154: See 2.14.

**All grammatical edits (2.2 – 2.15) suggested by the reviewer have been corrected. Furthermore, an experienced English proof reader has reviewed the manuscript and made additional edits for readability.**
